# *Zanthoxylum ailanthoides* Suppresses Oleic Acid-Induced Lipid Accumulation through an Activation of LKB1/AMPK Pathway in HepG2 Cells

**DOI:** 10.1155/2018/3140267

**Published:** 2018-01-08

**Authors:** Eun-Bin Kwon, Myung-Ji Kang, Soo-Yeon Kim, Yong-Moon Lee, Mi-Kyeong Lee, Heung Joo Yuk, Hyung Won Ryu, Su Ui Lee, Sei-Ryang Oh, Dong-Oh Moon, Hyun-Sun Lee, Mun-Ock Kim

**Affiliations:** ^1^Korea Research Institute of Bioscience and Biotechnology (KRIBB), Cheongju, Chungbuk 28116, Republic of Korea; ^2^College of Pharmacy, Chungbuk National University, Cheongju, Chungbuk 28644, Republic of Korea; ^3^Department of Biology Education, Daegu University, Gyeongsan-si, Gyeongsangbuk 38453, Republic of Korea

## Abstract

*Zanthoxylum ailanthoides* (ZA) has been used as folk medicines in East Asian and recently reported to have several bioactivity; however, the studies of ZA on the regulation of triacylglycerol (TG) biosynthesis have not been elucidated yet. In this study, we examined whether the methanol extract of ZA (ZA-M) could reduce oleic acid- (OA-) induced intracellular lipid accumulation and confirmed its mode of action in HepG2 cells. ZA-M was shown to promote the phosphorylation of AMPK and its upstream LKB1, followed by reduction of lipogenic gene expressions. As a result, treatment of ZA-M blocked de novo TG biosynthesis and subsequently mitigated intracellular neutral lipid accumulation in HepG2 cells. ZA-M also inhibited OA-induced production of reactive oxygen species (ROS) and TNF-*α*, suggesting that ZA-M possess the anti-inflammatory feature in fatty acid over accumulated condition. Taken together, these results suggest that ZA-M attenuates OA-induced lipid accumulation and inflammation through the activation of LKB1/AMPK signaling pathway in HepG2 cells.

## 1. Introduction

Nonalcoholic fatty liver disease (NAFLD), defined by a hepatic TG content exceeding 5% of liver weight, is one of the most common causes of chronic liver disease. The prevalence of NAFLD continues to increase with the growing obesity epidemic approximately 30% of the world population [1]. NAFLD accompanies various hepatic diseases ranging from simple steatosis to nonalcoholic steatohepatitis (NASH), fibrosis, cirrhosis, and hepatocarcinoma [2, 3]. In addition, NAFLD is associated with insulin resistance and hypertriglyceridemia and, more generally, with the metabolic syndrome [4]. Development of agent that can alleviate hepatic lipid accumulation may be one of the therapeutic approaches to treatment of NAFLD and associated hepatic disorders.

AMPK, an energy-sensing protein complex, is activated in response to an increase in the AMP:ATP ratio during hypoxia or starvation and upstream kinases including the tumor-suppressor liver kinase B1 (LKB1); the calcium-dependent calcium/calmodulin-dependent protein kinase kinase *β* (CaMKK*β*) [5–7]. Activated AMPK suppresses cleavage processing of sterol regulatory element-binding protein-1c (SREBP-1c) and de novo lipogenesis and stimulates fatty acid oxidation, glucose production, and protein synthesis in the liver [8]. AMPK activators, including metformin and thiazolidinediones (TDZs), have been shown to reduce the hepatic steatosis [9]; however, their use may be associated with several adverse effects. Commonly reported side effects of metformin include lactic acidosis, diarrhea, nausea, and vomiting. TDZs are usually well tolerated and induce water retention leading to edema and coronary heart disease. It is obvious that AMPK is one of the promising therapeutic targets in the treatment of NAFLD. There is a need to attempt to develop the new drugs with low side effects.

ZA is a medium to large tree with odd, pinnate leaves and conical spines in the main stem, distributed in places like China, Japan, and Korea. Leaves and bark of ZA are used as folk medicines to allaying pain and insecticide in Korea. The identified constituents of this plant are such as benzo[c]phenanthridines, quinolines, coumarins, flavonoids, lignans, amides, and terpenoids [10], and some compounds have been shown to have antiplatelet aggregation [11], anti-HIV [12], anti-oxidant [13, 14], anti-cancer [10, 15], and anti-inflammatory [16, 17] activities. Currently, the protective effects of ZA-M on free fatty acid-induced hepatocyte lipid accumulation were not characterized. The studies on* in vitro* cell models of hepatic steatosis largely use OA to induce fat deposition in hepatocytes [18, 19]. Therefore, this study was designed to investigate the effect of ZA-M on OA-induced cellular hepatic steatosis and to reveal its mode of action in HepG2 cells.

## 2. Materials and Methods

### 2.1. Chemicals and Reagents

3-[4,5-Dimethylthiazol-2-yl]-2,5 diphenyl tetrazolium bromide (MTT), dimethyl sulfoxide (DMSO), OA, Oil Red O, and anti-GPAT antibody were purchased from Sigma-Aldrich Co. (St. Louis, MO, USA). Dulbecco's modified eagle's medium (DMEM) was purchased from WelGENE Inc. (Daegu, Korea). Fetal Bovine Serum (FBS), Antibiotic-Antimycotic, and TRIzol reagent were purchased from Gibco-Invitrogen (Grand Island, NY, USA). BODIPY493/503, Hoechst 33342, and enhanced chemiluminescence (ECL kit) were purchased from Thermo Fisher Scientific Inc. (Waltham, MA, USA). Bradford reagents required for protein quantification were obtained from Bio-Rad (Richmond, CA). Antibodies against AMPK*α*/*β*, phosphorylated AMPK*α*/*β* (p-AMPK) (Thr172), acetyl-CoA carboxylase (ACC), phosphorylated ACC (p-ACC) (Ser79), liver kinase B1 (LKB1), phosphorylated LKB1 (p-LKB1) (Ser428), and tumor necrosis factor alpha (TNF-*α*) were obtained from Cell Signaling Technology, Inc. (Danvers, MA, USA). Antibodies against SREBP-1C, fatty acid synthesis (FAS), diglyceride acyltransferase 1/2 (DGAT1/2), stearoyl-CoA desaturase-1 (SCD1), and *β*-actin were obtained from Santa Cruz Biotechnology (CA, USA).

### 2.2. Plant Material and Extracts

Branches and leaves of ZA were collected in September 2014 from Jeju Island, Korea, and was identified by a botanist, Professor K. H. Bae (College of Pharmacy, Chungnam National University, Daejeon, Korea). Its voucher specimen (herbarium number 010-035) has been preserved at the Korea Plant Extract Bank (Korea Research Institute of Bioscience and Biotechnology, Daejeon, Korea). The dried plant material (3 kg) was wholly extracted with MeOH by maceration for 2 weeks at room temperature. The extract was concentrated at 40°C under reduced pressure to obtain the crude ZA-M. The extract was sealed and stored in a dark at −20°C.

### 2.3. Cell Culture

Human hepatocellular carcinoma HepG2 cells were obtained from American Type Culture Collection (ATCC, USA). Cells were cultured in DMEM supplemented with 10% FBS and 1% Antibiotic-Antimycotic agent in an incubator under an atmosphere of 5% CO_2_ at 37°C.

### 2.4. Cell Viability

HepG2 cells (5 × 10^5^ cells/ml) were seeded in 24-well plates. ZA-M was dissolved in DMSO to make a stock solution of 50 mg/ml and serially diluted to obtain final concentration of 10, 30, and 50 *μ*g/ml. Sodium oleate was dissolved in DW and prepared as it is warmed in 56°C water bath until the solution is clear. Cells were treated with ZA-M for 2 or 24 h, and cell viability was measured using MTT assay. The MTT assay is based on the conversion of MTT into insoluble formazan precipitate by mitochondrial dehydrogenases present only in viable cells. The formazan crystals were dissolved in 1 ml DMSO and the absorbance at 540 nm was measured with a microplate reader (Epoch, Biotek, USA).

### 2.5. Staining of Lipid Droplets with Oil Red O and BODIPY 493/503

HepG2 cells (5 × 10^5^ cells/ml) were pretreated with various concentrations of ZA-M for 1 h before exposure of 500 *μ*M OA for 24 h. After incubation, cells were fixed with 4% paraformaldehyde and stained with working solution of Oil Red O for 10 min at room temperature. After several washings, cells were observed under a light microscope. To quantify Oil Red O content, 100% isopropanol was added to each sample, which was read using a microplate reader at 500 nm. The cells were incubated for 15 min with Hoechst 33342 to stain nuclei and Bodipy 493/503 to stain neutral lipids and observed under a fluorescent microscope (Nikon, Japan).

### 2.6. RNA Isolation and RT-PCR Analysis

Total RNA was extracted with the TRIzol reagent, according to the manufacturer's instructions. 5 *μ*g RNA from each sample was converted into first-stranded DNA by an ImProm-II Reverse Transcriptase (Promega, Madison, WI, USA). RT-PCR was performed on a SureCycler 8800 (Agilent Technologies, Santa Clara, USA). PCR reactions were performed in a total volume of 20 *μ*L comprising 2 *μ*L of cDNA product, 0.2 mM of each dNTP, 20 pmol of each primer, and 0.8 units of Taq polymerase. Primer sequences for glyceraldehyde phosphate dehydrogenase (GAPDH), SREBP-1c, FAS, GPAT1, DGAT1, DGAT2, SCD1, and TNF-*α* were performed as described in Table S1. Final PCR products were visualized on 1% agarose gels stained with Noble view (Noble Bio, Suwon, South Korea). Densitometry quantification was performed using Multi-Gauge software (Fujifilm, Japan) and marked with a number after calculating the relative band intensities compared to GAPDH gene expression.

### 2.7. Western Blot Analysis

HepG2 cells (5 × 10^5^ cells/ml) were pretreated with various concentrations of ZA-M (10, 30, and 50 *μ*g/ml) for 1 h before exposure of 500 *μ*M OA for 24 h. After incubation, cells were lysed with Pro-Prep (Intron Biotechnology, Seoul, South Korea) on ice for 30 min. After centrifugation (13,200 ×g, for 25 min at 4°C), the supernatant was collected, and protein concentrations were determined using a Bradford method. Equal amounts of cell extracted protein were separated by SDS-PAGE electrophoresis and transferred to polyvinylidene difluoride membrane (Merck Millipore, Billerica, MA, USA). After being blocked with blocking solution, blots were incubated with antibodies and detection undertaken with an enhanced chemiluminescence reagent (Thermo Scientific, Rockford, IL, USA). Densitometry quantification was performed using Multi-Gauge software and marked with a number after calculating the relative band intensities compared to *β*-actin expression.

### 2.8. Determination of De Novo Synthesized TG

HepG2 cells (5 × 10^5^ cells/ml) were incubated with various concentrations of ZA-M in the presence of [^14^C]-glycerol (0.6 *μ*Ci) for 6 h. At the end of the incubation, intracellular lipids were extracted with a mixture of hexane: isopropanol (3 : 2, v/v) and separated on a TLC plate using a hexane: diethyl ether: acetic acid (80 : 20 : 1, v/v/v) solution as a developing solvent. The isotope-labeled TGs were detected and quantified with a bioimaging analyzer (FLA-7000, Fujifilm, Japan).

### 2.9. Determination of TG Content

After incubation, cells were lysed with 5% NP-40. Cellular TG was measured by an enzymatic colorimetric method with the TG assay kit (Bioassay systems, CA, USA) as per the manufacturer's instructions.

### 2.10. AMPK Activity Assay

AMPK activity was determined using the CycLex AMPK Kinase Assay Kit (CycLex Co., Ltd., Nagano, Japan) according to the manufacturer's instructions. Change of the chromogenic substrate tetramethylbenzidine was quantitated by absorbance measurement at 450 nm.

### 2.11. Flow Cytometric Analysis

Intracellular neutral lipids and ROS (O_2_^−^ and H_2_O_2_) were measured using Bodipy 493/503, hydroethidine (HE), and 2′7′-dichlorofluorescein diacetate (H_2_DCF-DA) in HepG2 cells. Bodipy 493/503 (10 *μ*g/ml), HE (1 *μ*M), or H_2_DCF-DA (5 *μ*M) was loaded for 30 minutes before harvesting. The cells were then washed and analyzed by flow cytometry (FACSCalibur, Becton Dickinson, CA, USA).

### 2.12. Determination of TNF-*α* Levels

The TNF-*α* levels in the culture medium were determined by TNF-*α* ELISA kit (R&D Systems, MN, USA) according to the manufacturer's instructions.

### 2.13. Statistical Analysis

The data are presented as the mean ± standard deviation (SD). One-way ANOVA followed by Dunnett's multiple comparison test was used for overall experiments. A value of *p* <  0.05 was considered statistically significant.

## 3. Results

### 3.1. Effect of ZA-M and OA on Cell Viability

**Figure 1:**
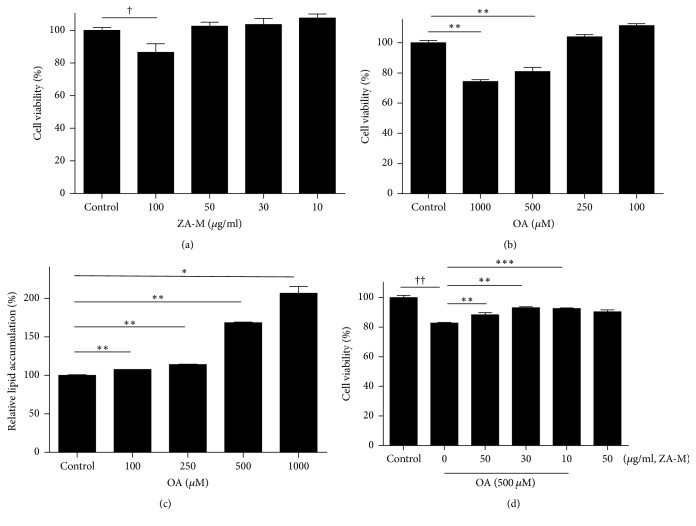
*Effect of ZA-M and OA on HepG2 cell viability*. (a and b) HepG2 cells were treated with various concentrations of ZA-M (10, 30, 50, and 100 *μ*g/ml) and OA (100, 250, 500, and 1000 *μ*M) for 24 h, respectively. Then, MTT assay was performed. (c) The cells were exposed to different concentrations of OA for 24 h, followed by Oil Red O staining to determine the lipid accumulation. (d) The cells were pretreated with indicated concentrations of ZA-M for 1 h, then exposed to 500 *μ*M OA for 24 h. Cell viability was measured by MTT assay. The bar graphs show the mean ± SD of 3 independent experiments (^†^*p* < 0.05 and ^††^*p* < 0.01 compared with the DMSO control; ^*∗*^*p* < 0.05, ^*∗∗*^*p* < 0.01, and ^*∗∗∗*^*p* < 0.001 compared with the OA treated control).

To evaluate the effects of ZA-M and OA on the cell viability of HepG2 cells, MTT assay was performed. As shown in Figure 1(a), the data indicate that concentrations of 10~50 *μ*g/ml ZA-M are not cytotoxic to HepG2 cells. ZA-M treatment with 100 *μ*g/ml for 24 h resulted in a slight inhibition of cell growth. In our result, OA reduced cell viability over the 500 *μ*M concentration (approximately 80% versus control group, Figure 1(b)), however, starting with 500 *μ*M OA exposure induced lipid accumulation (>150% versus vehicle control) in our experiment condition (Figure 1(c)). These results show that cell growth is retarded during excessive lipid accumulation. The pretreatment of ZA-M before 500 *μ*M OA exposure slightly restored cell viability with concentration-dependent manner compared with OA treatment only (Figure 1(d)). Therefore, we determined the optical range of ZA-M concentrations to be 10, 30, and 50 *μ*g/ml and OA to be 500 *μ*M during this study.

### 3.2. ZA-M Inhibits OA-Induced Lipid Accumulation in HepG2 Cells

**Figure 2:**
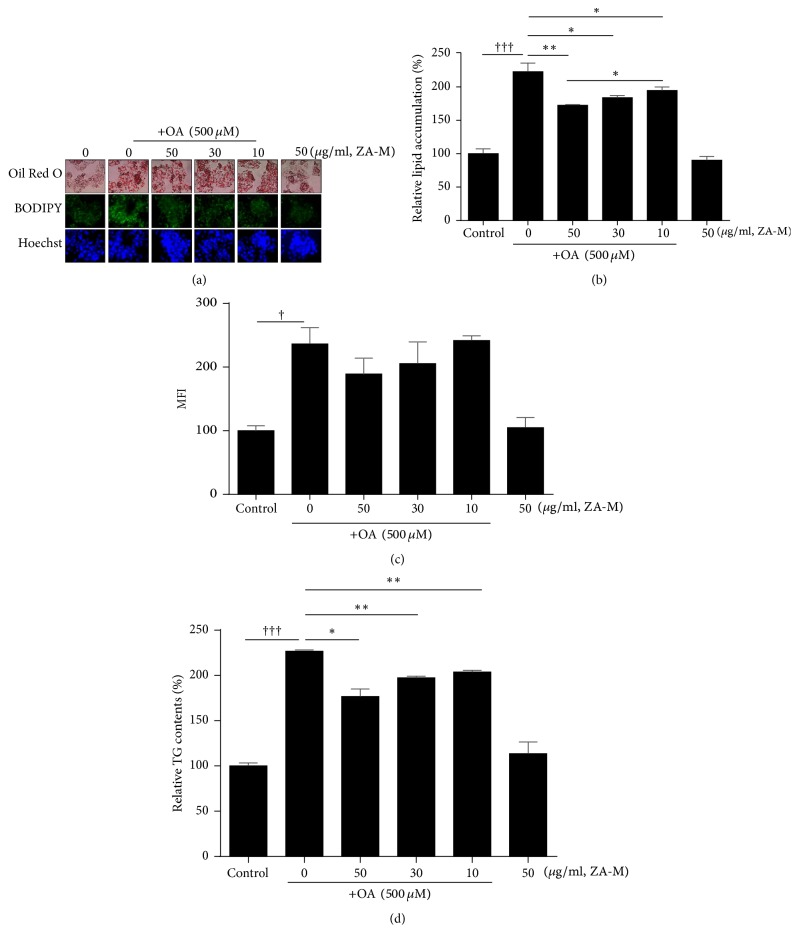
*Effect of ZA-M on OA-induced intracellular lipid accumulation in HepG2 cells*. (a) The cells were pretreated with indicated concentrations of ZA-M for 1 h, followed by exposed to 500 *μ*M OA for 24 h. Lipid accumulation was determined by using Oil Red O and BODIPY493/503 staining. Nuclei were counterstained with Hoechst 33342 dye. (b) Quantification of intracellular lipid accumulation. Total lipids stained with Oil Red O were extracted in absolute isopropanol, after which the absorbance of the solution was measured at 500 nm. (c) Quantitative analysis was performed by flow cytometry after BODIPY493/503 staining. (d) Quantification of TG contents by using commercial kit. The bar graphs show the mean ± SD of 3 independent experiments (^†††^*p* < 0.001 compared with the DMSO control; ^*∗*^*p* < 0.05 and ^*∗∗*^*p* < 0.01 compared with the OA treated control).

In order to determine whether ZA-M can reduce the lipid accumulation in HepG2 cells, we stained the cells using Oil Red O and BODIPY 493/503 to detect the intracellular neutral lipid content. Cells were treated with various concentrations of ZA-M for 1 h before OA treatment for 24 h. As shown in Figure 2(a), the decrease in color or fluorescent intensity of the dyeing reagent presents that the intracellular lipid content was significantly reduced by the pretreatment of ZA-M compared with OA alone. The quantitative data of Oil Red O displayed 12.6%, 17.5%, and 22.5% reduction of lipid contents by ZA-M treatment of 10, 30, and 50 *μ*g/ml, respectively, compared with OA alone (Figure 2(b)). Because BODIPY 493/503 more specifically distinguishes neutral lipids from other phospholipids or amphipathic lipids than Oil Red O, lipid droplets were stained using BODIPY 493/503 (green) and nuclei using Hoechst 33342 (blue) (Figure 2(a), middle and bottom lane). These results were further quantified using flow cytometry. Mean fluorescence intensity (MFI) was decreased by 3%, 9.9%, and 18.8% by ZA-M treatment at concentrations of 10, 30, and 50 *μ*g/ml, respectively, compared with OA alone (Figure 2(c)). As expected, intracellular TG quantification by commercially available kit showed a similar tendency to the previous results (Figure 2(d)). Collectively, these data confirm suggest that ZA-M could prevent OA-induced lipid accumulation in HepG2 cells.

### 3.3. ZA-M Inhibits the Lipogenic Gene Expression

**Figure 3:**
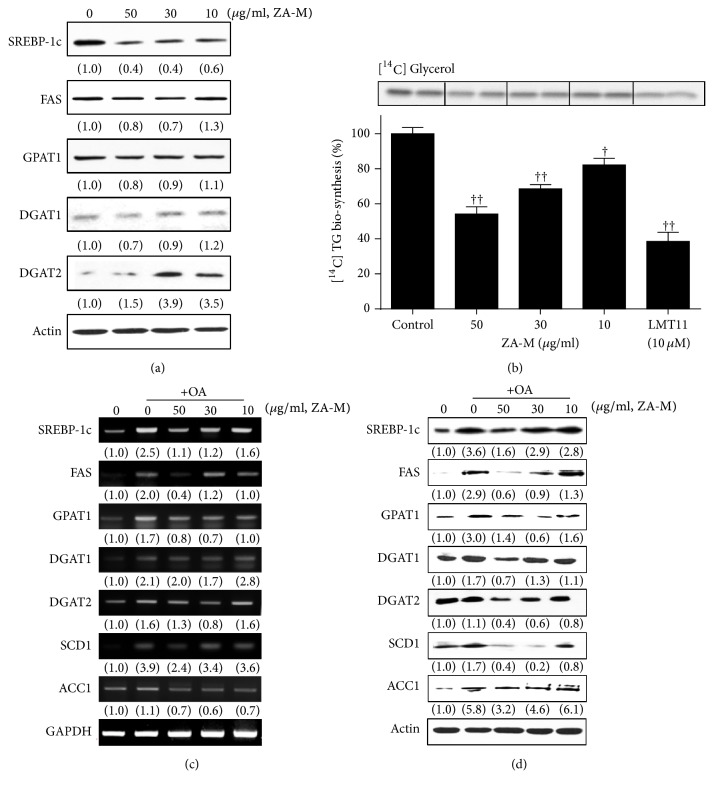
*Effect of ZA-M on transcriptional/translational expression of lipogenic genes and sequential TG biosynthesis in HepG2 cells*. (a) Western blot analysis of lipogenic protein expression level was performed after treatment of indicated concentrations of ZA-M (0, 50, 30, and 10 *μ*g/ml) in HepG2 cells. (b) De novo TG biosynthesis. The cells were cotreated with various concentrations of ZA-M and [^14^C] glycerol for 6 h, and then TLC-based analysis of lipid intermediates was performed. Each [^14^C] TG band was quantified by using the Multi-Gauge V3.0 software (Fujifilm). The relative activity was calculated by setting the value from DMSO-treated cells to 100%. (c and d) Effect of ZA-M on OA-induced upregulation of transcriptional/translational expression of lipogenic genes was analyzed RT-PCR and western blot, respectively. The bar graphs show the mean ± SD of 3 independent experiments (^†^*p* < 0.05 and ^††^*p* < 0.01 compared with the DMSO control).

In the process of identifying the mechanism of ZA-M involved in the inhibition of hepatic lipid accumulation, we presumed that ZA-M could regulate the expression of lipogenic genes. As expected, the treatment of ZA-M alone for 24 h decreased SREBP-1c, a key transcription factor that regulate lipogenesis, with dose-dependent manner in HepG2 cells (Figure 3(a)). Also, the expression of SREBP-1 target genes including FAS, GPAT1, DGAT1, and -2 was showed continuous decrease tendency. Next, we examined that whether ZA-M can actually reduce de novo lipogenesis in HepG2 cells. We traced the newly synthesized isotope-labeled TG by adding ^14^C-labeled glycerol serves as a substrate of TG biosynthesis. As observed in Figure 3(b), ZA-M decreased the incorporation of ^14^C-labeled glycerol into TG in HepG2 cells (inhibition of 17.8, 31.5 and 45.9* *% at 10, 30 and 50 *μ*g/ml ZA-M, respectively). It has been found that OA treatment increased the expressions of SREBP-1c, FAS, GPAT1, DGAT1, -2 and SCD1 at both protein and mRNA levels (Figures 3(c) and 3(d)). However, the pretreatment of ZA-M before OA exposure significantly attenuated the OA-induced expression of SREBP-c1 and its target genes. These data demonstrate that ZA-M modulates SREBP-1c and its downstream target genes, subsequently suppress do novo TG biosynthesis at the cellular level.

### 3.4. Effects of ZA-M on AMPK Activity in HepG2 Cells

**Figure 4:**
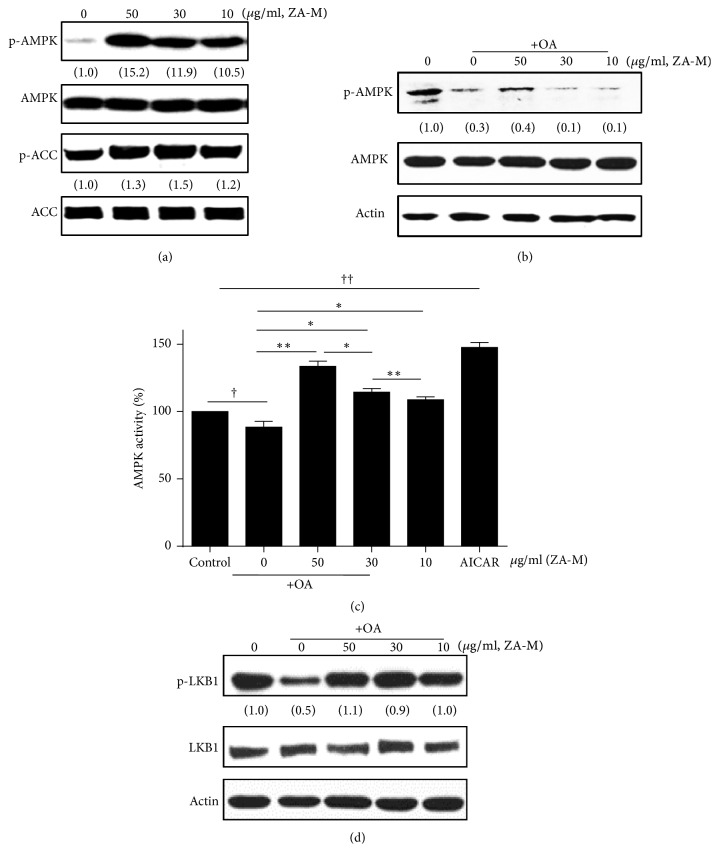
*ZA-M activates the LKB1/AMPK signaling pathway*. (a, b) Western blot analysis of phosphorylation status of AMPK (Thr 172) and ACC (Ser 79) after treatment of indicated concentrations of ZA-M (50, 30, and 10 *μ*g/ml) in the present or absent of OA in HepG2 cells. (c) AMPK kinase activity. (d) Western blot analysis of phosphorylation status of LKB-1 after treatment of indicated concentrations of ZA-M (50, 30, and 10 *μ*g/ml) in the present in HepG2 cells. The bar graphs show the mean ± SD of 3 independent experiments (^††^*p* < 0.01 and ^†††^*p* < 0.001 compared with the DMSO control; ^*∗*^*p* < 0.05 compared with the OA treated control).

Given that AMPK is a key regulator of lipogenesis, we next examined the effect of ZA-M on AMPK and its primary downstream enzyme acetyl-CoA carboxylase (ACC) in HepG2 cells. Cell were treated with various concentrations of ZA-M for 2 h. As observed in Figure 4(a), treatment of ZA-M increased the phosphorylation levels of both AMPK (Thr-172) and ACC (Ser-79) with concentration-dependent manner, compared with the control. In addition, OA exposure to HepG2 cells somewhat reduced phosphorylation of AMPK, but pre-treatment of 50 *μ*g/ml ZA-M has reversed the level of phosphorylation of AMPK similar to control level (Figure 4(b)). Next, we performed AMPK activity assay by the principle of detecting the phosphorylation of a synthetic AMPK substrate peptides. Addition of ZA-M in presence of OA significantly increased AMPK activity (Figure 4(c)). For these experiments, the AMP mimetic AICAR was used as a positive control for AMPK activation, and treatment of AICAR activates basal AMPK activity about 50% compared with control. Between the known upstream regulator of AMPK including LKB1 and CaMKK*β*, we first examined the phosphorylation of LKB1. Fortunately, ZA-M considerably reversed the OA-induced de-phosphorylated state of LKB1 (Figure 4(d)). Collectively, we suggest that ZA-M activates AMPK and reverses OA-induced suppression of LKB1/AMPK signaling pathway in HepG2 cells.

### 3.5. ZA-M Reduces ROS Generation

**Figure 5:**
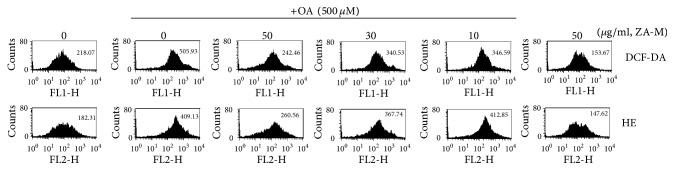
*Effect of ZA-M on OA-induced ROS production*. The cellular ROS level was measured by FACS using the H_2_DCFDA and HE probe. The numbers at the figure indicate the mean fluorescence intensity.

Increased fatty acid availability activates mitochondrial oxidation, leading to over-production of reactive oxygen species (ROS) [17], which in turn induces lipid peroxidation, protein denaturation and DNA damage. Furthermore, loading the excessive free fatty acids have been previously reported to generate ROS in various cells, such as pancreatic islet cells [20], hepatocytes [21], and adipocytes [22]. Intracellular ROS levels accompany certain pathological conditions such as insulin resistance and type 2 diabetes [20]. To evaluate the effect of ZA-M and OA on intracellular ROS production, cells were stained with DCFH-DA and HE fluorescent dye to detect hydrogen peroxide and superoxide anion, respectively. The substrate DCFH-DA is a stable nonpolar molecule that readily diffuses across the cell membrane and becomes highly fluorescent upon oxidized by hydrogen peroxide [23]. Intracellular superoxide can be measured by HE which is converted into red fluorescent via superoxide anion. As shown in Figure 5, 500 *μ*M OA increased the intracellular ROS generation more than twice both hydrogen peroxide and superoxide, respectively, compared with control. However, the elevated ROS levels caused by OA were markedly decreased by ZA-M treatment with concentration-dependent manner. Collectively, it is a possibility that ZA-M attenuates the mitochondrial oxidative stress since ZA-M alleviated the free fatty acid overloaded state.

### 3.6. ZA-M Decrease Hepatic Inflammation

**Figure 6:**
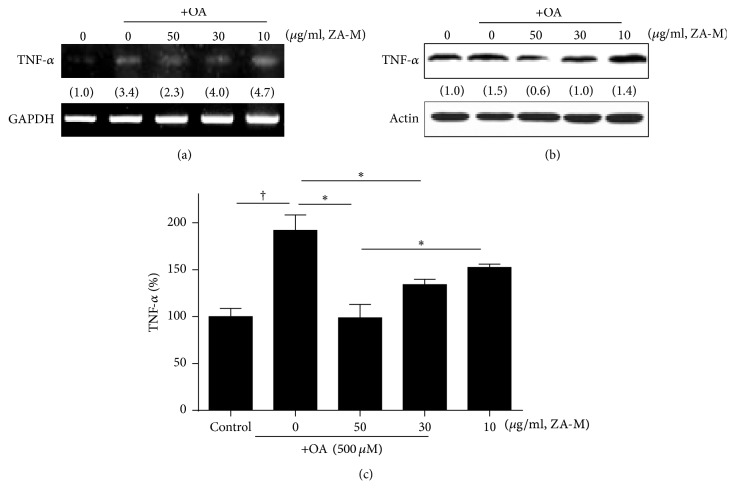
*Effect of ZA-M on OA-induced TNF-α production*. (a and b) Expression of TNF-*α* was confirmed by RT-PCR and western blot, respectively. (c) The cell-free supernatants were collected and analyzed for TNF-*α* production by ELISA. The bar graphs show the mean ± SD of 3 independent experiments (^†^*p* < 0.05 compared with the DMSO control; ^*∗*^*p* < 0.05 compared with the OA treated control).

Since TNF-*α*, a mediator of inflammation, plays a major role in the pathogenesis of NAFLD and development of insulin resistance and impaired glucose tolerance [24], we determined the effect of ZA-M on OA-induced production of TNF-*α*. The mRNA and protein expression levels of TNF-*α* increased dramatically after OA treatment; however, ZA-M prevented the OA-induced upregulation of TNF-*α* in a dose-dependent manner (Figures 6(a) and 6(b)). Finally, we quantified the secreted TNF-*α* in cell culture medium by commercial ELISA kit (Figure 6(c)). Cells released about 2-fold amount of TNF-*α* after OA treatment. ZA-M significantly reduced the OA-stimulated extracellular TNF-*α* dose-dependently. As a result of this, ZA-M could ameliorate proinflammatory response in OA-induced cellular steatosis model.

## 4. Discussion

AMPK can have a multitude effects on various tissues. The activation of AMPK results in fatty acid oxidation in muscle and liver; the inhibition of hepatic glucose production, cholesterol and TG synthesis, and lipogenesis; and the stimulation of glucose uptake in muscle [25–27]. Therefore, AMPK is an attractive target for metabolic disorder therapies due to its role as a master metabolic regulator. The activation of AMPK requires both an increase in the intracellular AMP : ATP ratio and phosphorylation of Thr172 of the *α*-subunit by one of its three upstream kinases: LKB1 [5], CaMKK*β* [28], or transforming growth factor-*β* activated protein kinase-1 (TAK1) [29]. In our results, western blot analysis revealed that LKB1 is the upstream molecule of ZA-M-induced AMPK activation (Figure 4). Unfortunately, direct evidence of molecular mechanism underlying activation of AMPK by ZA-M has not been demonstrated in this study, and further study is needed. In our study, OA-induced dephosphorylation of AMPK represents the cell status that switches on to lipogenesis. The treatment of ZA-M reversed OA-induced inactivation of AMPK in a dose-dependent manner. Upon activation, AMPK phosphorylates its downstream targets, a main one being ACC at Ser79 (an inhibitory site), which allows long-chain FAs to enter the mitochondria for oxidation [30]. We confirmed the effect of ZA-M on AMPK activation in three ways: checking the relative phosphorylation status of AMPK and its downstream target ACC (Figure 4(a)) and its kinase activity (Figure 4(c)).

Because the activated AMPK results in fatty acid synthesis via a main transcription factor SREBP1c, we carried out western blot analysis to confirm the expression level of SREBP1c and its target genes. As a result, the both transcriptional and translational levels of SREBP1c and its target genes were gradually reduced on the treatment of ZA-M alone as well as in the OA-induced experimental condition. Consequently, we suggest that ZA-M inhibited OA-induced intracellular lipid accumulation through activation of LKB1/AMPK pathway in HepG2 cells.

Numerous physiological, pharmacological, hormone, and natural activators of AMPK are known [5]. Several AMPK activator currently used T2D medications, such as metformin and TZDs, indirectly activate AMPK; however, no direct AMPK activators reach clinic owing to poor pharmacokinetic profiles, off-target effects, and so on [31]. Nonetheless, AMPK is a still attractive target because of its physiological role and in that of decreased AMPK activity in tissues such as in the muscle and adipose tissue of obese or insulin-resistant animals and humans [29, 31].

In normal subjects, fatty acid oxidation occurs in the mitochondrial through *β*-oxidation. However, mitochondrial *β*-oxidation is saturated in NAFLD underlying increased fatty acid availability, patients with NAFLD reported the defective mitochondrial functions, for example, reduced mitochondrial respiratory chain activity [32] and impaired ATP synthesis [33]. Electron leakage occurs from the interrupted electron transport chain because free FAs can act as specific complex I-directed inhibitors [34], which produces the ROS such as superoxide anion radical and hydrogen peroxide [35, 36]. As shown in Figure 5, the treatment of OA to HepG2 cells for 24 h drastically increased both production of the superoxide anion radical and hydrogen peroxide. Pretreatment of ZA-M abolished OA-induced intracellular ROS generation in HepG2 cells in a dose-dependent manner.

TNF-*α* is another considerable factor in the pathogenesis of mitochondrial dysfunction. As previous data, high blood TNF-*α* levels have been found in patients with NASH [24]. TNF-*α* concentrations in liver tissue in* ob/ob* mice were some 20-fold higher than in normal mice [37]. TNF-*α* inducing swelling of the mitochondria causes a bursting of the mitochondrial membrane leading to an interference between mitochondrial respiratory chain complexes I and III [38]. It has been reported that anti-TNF-*α* treatment in* ob/ob* mice improve complex I, II, III, and V activity, *β*-oxidation activity, and liver histology [37]. In our results, ZA-M markedly reduced OA-induced TNF-*α* expression at both transcriptional and translational level. Taken together, these results raised the therapeutic values of ZA-M on excessive free fatty acid-induced inflammation.

## 5. Conclusion

The present study reveals that ZA-M significantly reduces the neutral lipid level of OA-induced hepatic steatosis cellular model. The proven mechanism of ZA-M on decreasing intracellular lipid is the activation of LKB1/AMPK signaling pathway; therefore, we suggest that ZA-M attenuates the overloaded fatty acid-induced intracellular TG accumulation and inflammation in HepG2 cells.

## Abbreviations

ACC: Acetyl-CoA carboxylase
AMPK: AMP-activated protein kinase
LDs: Lipid droplets
LKB1: Liver kinase B1
MFI: Mean fluorescence intensity
NAFLD: Nonalcoholic fatty liver disease
NASH: Nonalcoholic steatohepatitis
OA: Oleic acid
ROS: Reactive oxygen species
SREBP-1c: Sterol regulatory element-binding protein-1c
TDZs: Thiazolidinediones
TNF-*α*: Tumor necrosis factor-*α*
TG: Triacylglycerol
ZA-M: The methanol extract of* Zanthoxylum ailanthoides*.

## Supplementary Materials

Supplementary 1 Figure S1: effect of ZA-M on OA-induced iNOS and COX-2 mRNA expression. Figure S2: densitometric analysis of the western blots and qPCR bands using Multi-Gauge software. Relative quantification (A) for Figure  3(a); (B) for Figure  3(c); (C) for Figure  3(d); (D) for Figure  4(a); (E) for Figures  4(b) and 4(d); and (F) for Figure  6(b).

Supplementary 2 Table S1: the primer sequences for reverse transcriptase-PCR.

## References

[1] Schattenberg J. M., Schuppan D. (2011). Nonalcoholic steatohepatitis: The therapeutic challenge of a global epidemic. *Current Opinion in Lipidology*.

[2] Katsiki N., Mikhailidis D. P., Mantzoros C. S. (2016). Non-alcoholic fatty liver disease and dyslipidemia: An update. *Metabolism - Clinical and Experimental*.

[3] Cusi K. (2016). Treatment of patients with type 2 diabetes and non-alcoholic fatty liver disease: current approaches and future directions. *Diabetologia*.

[4] Tessari P., Coracina A., Cosma A., Tiengo A. (2009). Hepatic lipid metabolism and non-alcoholic fatty liver disease. *Nutrition, Metabolism & Cardiovascular Diseases*.

[5] Kahn B. B., Alquier T., Carling D., Hardie D. G. (2005). AMP-activated protein kinase: ancient energy gauge provides clues to modern understanding of metabolism. *Cell Metabolism*.

[6] Viollet B., Horman S., Leclerc J. (2010). AMPK inhibition in health and disease. *Critical Reviews in Biochemistry and Molecular Biology*.

[7] Viollet B., Guigas B., Leclerc J. (2009). AMP-activated protein kinase in the regulation of hepatic energy metabolism: From physiology to therapeutic perspectives. *Acta Physiologica*.

[8] Wang S., Moustaid-Moussa N., Chen L. (2014). Novel insights of dietary polyphenols and obesity. *The Journal of Nutritional Biochemistry*.

[9] Zhou G., Myers R., Li Y. (2001). Role of AMP-activated protein kinase in mechanism of metformin action. *The Journal of Clinical Investigation*.

[10] Chung C.-Y., Hwang T.-L., Kuo L.-M. (2013). New benzo[c]phenanthridine and benzenoid derivatives, and other constituents from Zanthoxylum ailanthoides: Effects on neutrophil pro-inflammatory responses. *International Journal of Molecular Sciences*.

[11] Hsiao G., Chang C.-Y., Shen M.-Y. (2005). *α*-Naphthoflavone, a potent antiplatelet flavonoid, is mediated through inhibition of phospholipase C activity and stimulation of cyclic GMP formation. *Journal of Agricultural and Food Chemistry*.

[12] Chen J.-J., Chung C.-Y., Hwang T.-L., Chen J.-F. (2009). Amides and benzenoids from Zanthoxylum ailanthoides with inhibitory activity on superoxide generation and elastase release by neutrophils. *Journal of Natural Products*.

[13] Chu C.-Y., Lee H.-J., Chu C.-Y., Yin Y.-F., Tseng T.-H. (2009). Protective effects of leaf extract of Zanthoxylum ailanthoides on oxidation of low-density lipoprotein and accumulation of lipid in differentiated THP-1 cells. *Food and Chemical Toxicology*.

[14] Chou S. T., Peng H. Y., Chang C. T. (2011). Zanthoxylum ailanthoides Sieb and Zucc. extract inhibits growth and induces cell death through G2/M-phase arrest and activation of apoptotic signals in colo 205 human colon adenocarcinoma cells. *Anticancer Reseach*.

[15] Chou S.-T., Chan H.-H., Peng H.-Y., Liou M.-J., Wu T.-S. (2011). Isolation of substances with antiproliferative and apoptosis-inducing activities against leukemia cells from the leaves of Zanthoxylum ailanthoides Sieb. & Zucc. *Phytomedicine*.

[16] Kim H. J., Jun J.-G., Kim J.-K. (2013). 2-(4-hydroxyphenyl)-5-(3-hydroxypropenyl)-7-methoxybenzofuran, a novel ailanthoidol derivative, exerts anti-inflammatory effect through downregulation of mitogen-activated protein kinase in lipopolysaccharide-treated RAW 264.7 cells. *Korean Journal of Physiology & Pharmacology*.

[17] Bullon P., Newman H. N., Battino M. (2014). Obesity, diabetes mellitus, atherosclerosis and chronic periodontitis: a shared pathology via oxidative stress and mitochondrial dysfunction?. *Periodontology 2000*.

[18] Lee M.-S., Kim J.-S., Cho S.-M., Lee S. O., Kim S.-H., Lee H.-J. (2015). Fermented Rhus verniciflua Stokes Extract Exerts an Antihepatic Lipogenic Effect in Oleic-Acid-Induced HepG2 Cells via Upregulation of AMP-Activated Protein Kinase. *Journal of Agricultural and Food Chemistry*.

[19] Kim J.-H., Kang S.-I., Shin H.-S. (2013). Sasa quelpaertensis and p-coumaric acid attenuate oleic acid-induced lipid accumulation in HepG2 cells. *Bioscience, Biotechnology, and Biochemistry*.

[20] Rodrigues Graciano M. F., Valle M. M. R., Kowluru A., Curi R., Carpinelli A. R. (2011). Regulation of insulin secretion and production of reactive oxygen species by free fatty acids in pancreatic islets. *Islets*.

[21] Gusdon A. M., Song K.-X., Qu S. (2014). Nonalcoholic fatty liver disease: pathogenesis and therapeutics from a mitochondria-centric perspective. *Oxidative Medicine and Cellular Longevity*.

[22] Han C. Y. (2016). Roles of reactive oxygen species on insulin resistance in adipose tissue. *Diabetes & Metabolism*.

[23] Macey M. G., Sangster J., Veys P. A., Newland A. C. (1990). Flow cytometric analysis of the functional ability of neutrophils from patients with autoimmune neutropenia. *Journal of Microscopy*.

[24] Garcia-Ruiz I., Rodriguez-Juan C., Diaz-Sanjuan T. (2006). Uric acid and anti-TNF antibody improve mitochondrial dysfunction in ob/ob mice. *Hepatology*.

[25] Schimmack G., DeFronzo R. A., Musi N. (2006). AMP-activated protein kinase: role in metabolism and therapeutic implications. *Diabetes, Obesity and Metabolism*.

[26] Hardie D. G. (2007). AMP-activated/SNF1 protein kinases: conserved guardians of cellular energy. *Nature Reviews Molecular Cell Biology*.

[27] Grahame Hardie D., Ashford M. L. J. (2014). AMPK: regulating energy balance at the cellular and whole body levels. *Physiology Journal*.

[28] Racioppi L., Means A. R. (2012). Calcium/calmodulin-dependent protein kinase kinase 2: roles in signaling and pathophysiology. *The Journal of Biological Chemistry*.

[29] Coughlan K. A., Valentine R. J., Ruderman N. B., Saha A. K. (2014). AMPK activation: a therapeutic target for type 2 diabetes?. *Diabetes, Metabolic Syndrome and Obesity: Targets and Therapy*.

[30] Russo G. L., Russo M., Ungaro P. (2013). AMP-activated protein kinase: a target for old drugs against diabetes and cancer. *Biochemical Pharmacology*.

[31] Paradies G., Paradies V., Ruggiero F. M., Petrosillo G. (2014). Oxidative stress, cardiolipin and mitochondrial dysfunction in nonalcoholic fatty liver disease. *World Journal of Gastroenterology*.

[32] Koliaki C., Roden M. (2013). Hepatic energy metabolism in human diabetes mellitus, obesity and non-alcoholic fatty liver disease. *Molecular and Cellular Endocrinology*.

[33] Schönfeld P., Wojtczak L. (2008). Fatty acids as modulators of the cellular production of reactive oxygen species. *Free Radical Biology & Medicine*.

[34] Garcia-Ruiz C., Colell A., Morales A., Kaplowitz N., Fernandez-Checa J. C. (1995). Role of oxidative stress generated from the mitochondrial electron transport chain and mitochondrial glutathione status in loss of mitochondrial function and activation of transcription factor nuclear factor-*κ*B: studies with isolated mitochondria and rat hepatocytes. *Molecular Pharmacology*.

[35] Hensley K., Kotake Y., Sang H. (2000). Dietary choline restriction causes complex I dysfunction and increased H_2_O_2_ generation in liver mitochondria. *Carcinogenesis*.

[36] Pessayre D. (2007). Role of mitochondria in non-alcoholic fatty liver disease. *Journal of Gastroenterology and Hepatology*.

[37] Sánchez-Alcázar J. A., Schneider E., Martínez M. A. (2000). Tumor necrosis factor-*α* increases the steady-state reduction of cytochrome b of the mitochondrial respiratory chain in metabolically inhibited L929 cells. *The Journal of Biological Chemistry*.

[38] Higuchi M., Proske R. J., Yeh E. T. H. (1998). Inhibition of mitochondrial respiratory chain complex I by TNF results in cytochrome c release, membrane permeability transition, and apoptosis. *Oncogene*.

