# Corrigendum to “*Zanthoxylum ailanthoides* Suppresses Oleic Acid-Induced Lipid Accumulation through an Activation of LKB1/AMPK Pathway in HepG2 Cells”

**DOI:** 10.1155/2019/3498219

**Published:** 2019-07-14

**Authors:** Eun-Bin Kwon, Myung-Ji Kang, Soo-Yeon Kim, Yong-Moon Lee, Mi-Kyeong Lee, Heung Joo Yuk, Hyung Won Ryu, Su Ui Lee, Sei-Ryang Oh, Dong-Oh Moon, Hyun-Sun Lee, Mun-Ock Kim

**Affiliations:** ^1^Korea Research Institute of Bioscience and Biotechnology (KRIBB), Cheongju, Chungbuk 28116, Republic of Korea; ^2^College of Pharmacy, Chungbuk National University, Cheongju, Chungbuk 28644, Republic of Korea; ^3^Department of Biology Education, Daegu University, Gyeongsan-si, Gyeongsangbuk 38453, Republic of Korea

In the article titled “*Zanthoxylum ailanthoides* Suppresses Oleic Acid-Induced Lipid Accumulation through an Activation of LKB1/AMPK Pathway in HepG2 Cells” [1], there was figure duplication in Figure 4, where the second panel (AMPK) in Figure 4(b) is the same as the last panel (Actin) in Figure 4(d). 

The authors explained that, at the beginning, Figures 4(b) and 4(d) were one figure consisting of p-AMPK band (first panel in Figure 4(b)) and Actin band (last panel in Figure 4(d)). AMPK band (the second panel in Figure 4(b)) did not exist, as it needed to be confirmed through a Western blot experiment. Then, the Western blot results of p-LKB 1 and LKB 1 bands, performed under the same experimental conditions, were added. At the same time, due to a mix-up, the AMPK band (the second panel in Figure 4(b)) and the Actin band (last panel in Figure 4(d)) were duplicated. They clarified they did not recognize this duplication and the figure was then subdivided into Figures 4(b) and 4(d) and Actin band (the last panel in Figure 4(b)) was added. Therefore, Figure 4 should be corrected as shown below.

## Supplementary Materials

Supplementary Materials The original Western blot bands of Figures 3 and 4.

## Figures and Tables

**Figure 4:**
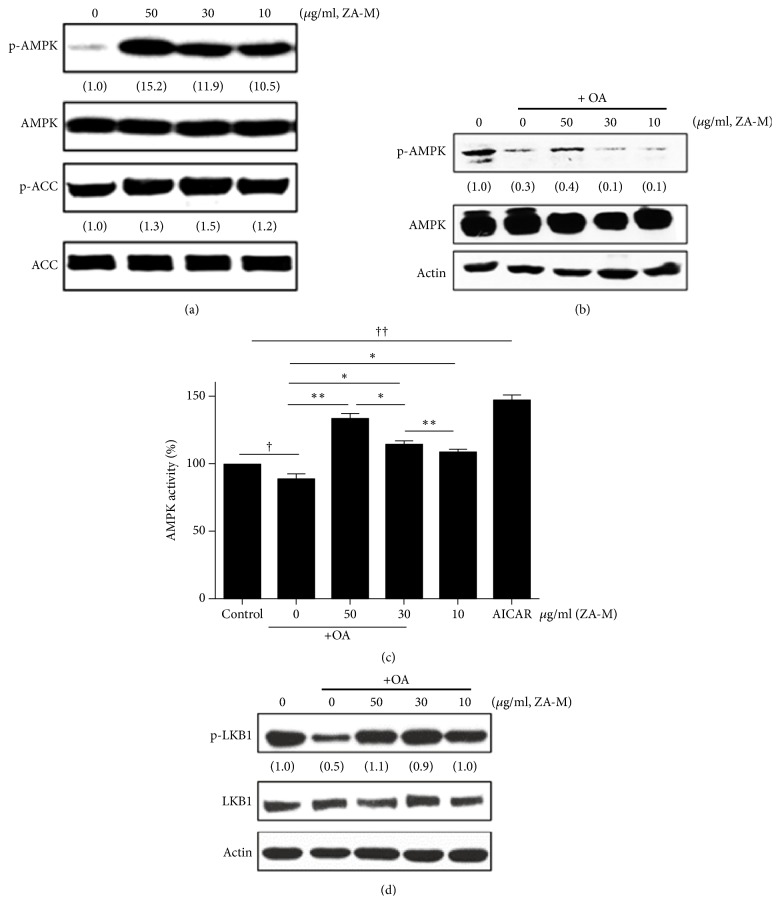
ZA-M activates the LKB1/AMPK signaling pathway. (a, b) Western blot analysis of phosphorylation status of AMPK (Thr 172) and ACC (Ser 79) after treatment of indicated concentrations of ZA-M (50, 30, and 10 *μ*g/ml) in the presence or absence of OA in HepG2 cells. (c) AMPK kinase activity. (d) Western blot analysis of phosphorylation status of LKB-1 after treatment of indicated concentrations of ZA-M (50, 30, and 10 *μ*g/ml) in the presence of HepG2 cells. The bar graphs show the mean ± SD of 3 independent experiments (^††^*p* < 0.01 and ^†††^*p* < 0.001 compared with the DMSO control; ^*∗*^*p* < 0.05 compared with the OA treated control).
