# Supplementary material for: Zanthoxylum ailanthoides Suppresses Oleic Acid-Induced Lipid Accumulation through an Activation of LKB1/AMPK Pathway in HepG2 Cells
Source: Evid Based Complement Alternat Med. 2018 Jan 8;2018:3140267. doi: 10.1155/2018/3140267 (PMC5817260; doi:10.1155/2018/3140267)
Supplement: Supplementary 1 — Figure S1: effect of ZA-M on OA-induced iNOS and COX-2 mRNA expression. Figure S2: densitometric analysis of the western blots and qPCR bands using Multi-Gauge software. Relative quantification (A) for Figure 3(a); (B) for Figure 3(c); (C) for Figure 3(d); (D) for Figure 4(a); (E) for Figures 4(b) and 4(d); and (F) for Figure 6(b). [file 3140267.f1.docx]

**Supplementary Figures**

**Figure S1.**


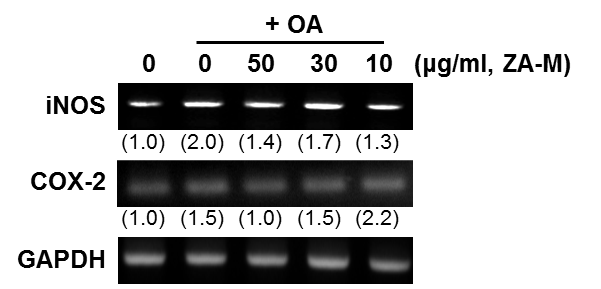


**Figure S1.** Effect of ZA-M on OA-induced iNOS and COX-2 mRNA expression.

**Figure S2.**

**(A)**

**
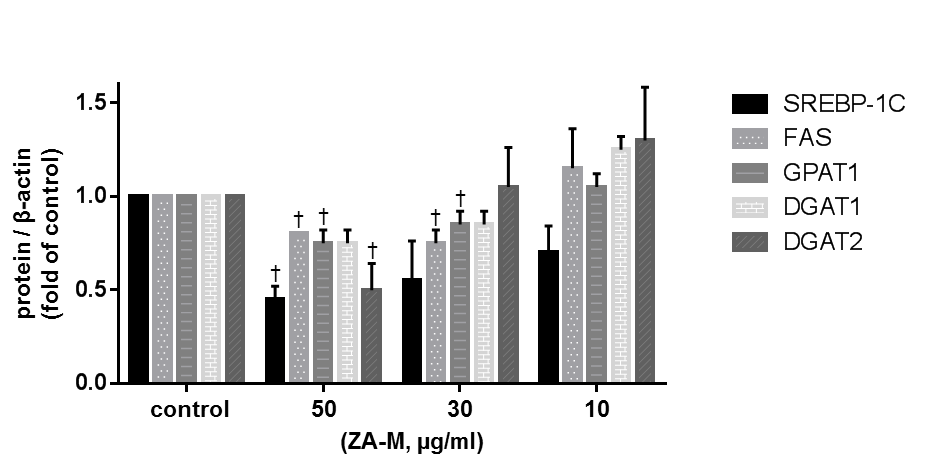
**

**(B)**

**
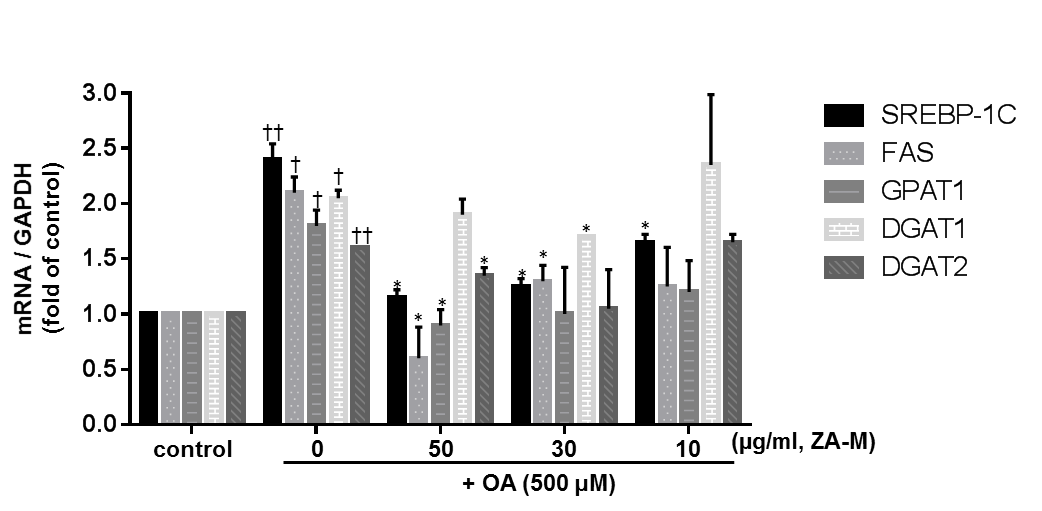
**

**(C)**

**
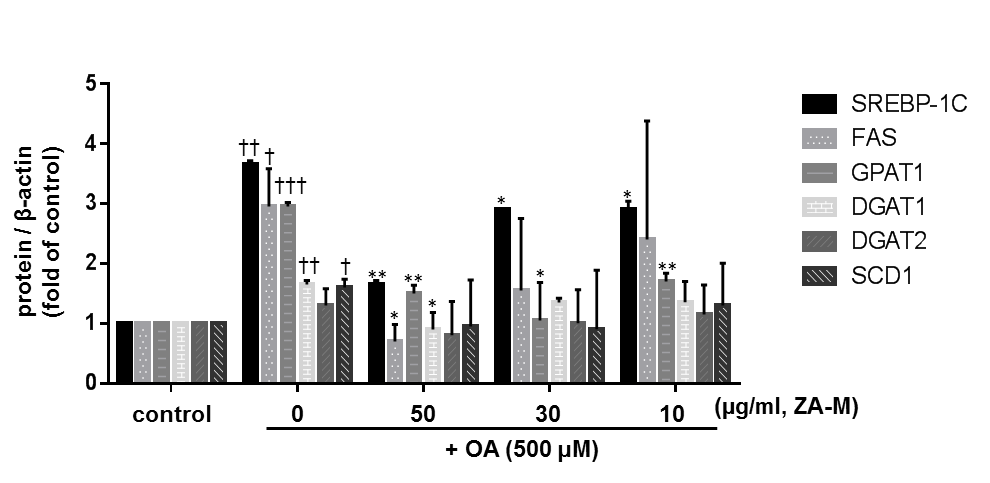
**

**(D)**

**
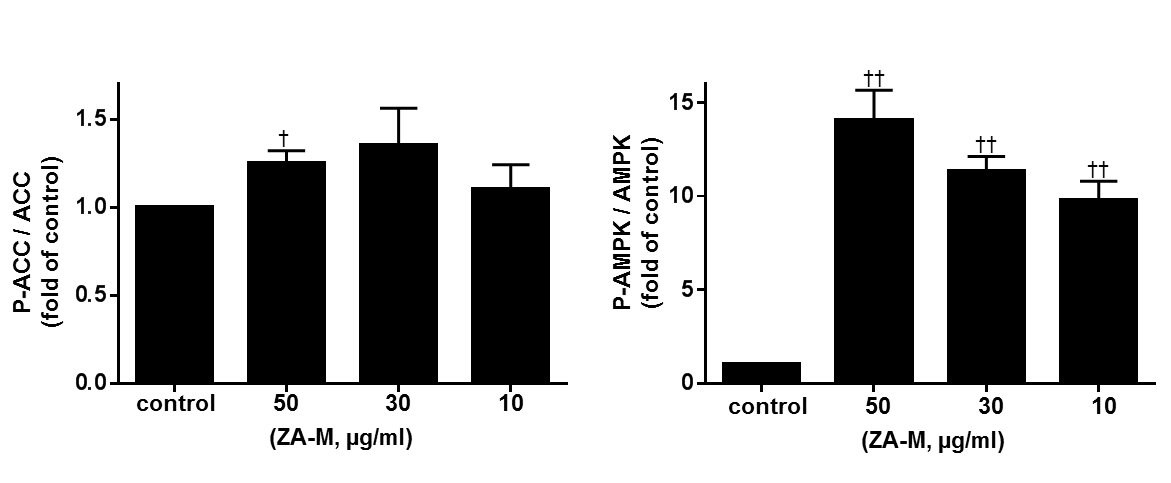
**

**(E)**

**
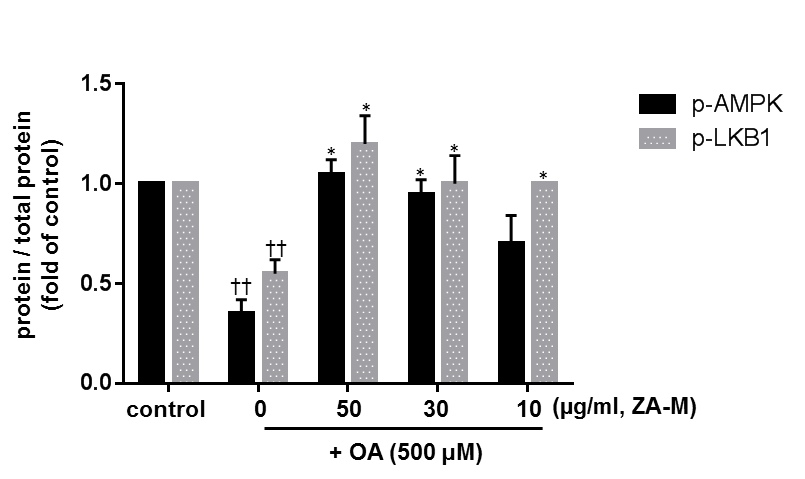
**

**(F)**

**
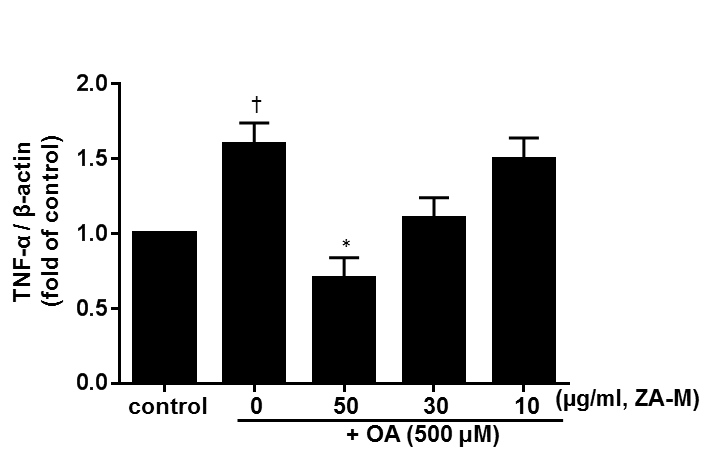
**

**Figure S2.** Densitometric analysis of the western blots and qPCR bands using MultiGauge software. Relative quantification (A) for the figure 3A, (B) for the figure 3C, (C) for the figure 3D, (D) for the figure 4A, (E) for the figures 4B and D, and (F) for the figure 6B.
