# Supplementary material for: Zanthoxylum ailanthoides Suppresses Oleic Acid-Induced Lipid Accumulation through an Activation of LKB1/AMPK Pathway in HepG2 Cells
Source: Evid Based Complement Alternat Med. 2018 Jan 8;2018:3140267. doi: 10.1155/2018/3140267 (PMC5817260; doi:10.1155/2018/3140267)
Supplement: Supplementary 2 — Table S1: the primer sequences for reverse transcriptase-PCR. [file 3140267.f2.docx]

| **Gene** | **Forward** | **Reverse** | **Annealing TM (°C)** | **Product size (bp)** |
| --- | --- | --- | --- | --- |
| ***srebp-1c*** | 5’-CCATGCTGGAACTGATGGAG-3’ | 5’-CTGAACTGTGTGACCCAGCC-3’ | 52 | 159 |
| ***fasn*** | 5’-TGGTCACGGACGATGACCGTCG-3’ | 5’-GCGGCAGTACCCATTCCCCGC-3’ | 50 | 578 |
| ***gpat1*** | 5’-GATCATTGCCGGTGAGACAG-3’ | 5’-CATAAGGGCATGTTTGCCAC-3’ | 55 | 207 |
| ***dgat1*** | 5’-CTGGTCCAGTCTTGGGGTCT-3’ | 5’-ACCAAGCTGGATAGATGGGG-3’ | 52 | 131 |
| ***dgat2*** | 5’-CCGACCACGATGATGATAGC-3’ | 5’-CCGACCACGATGATGATAGC-3’ | 52 | 173 |
| ***scd1*** | 5’-GGTGGTCACGAGCCCATTC-3’ | 5’-CCAACACAATGGCATTCCAG-3’ | 58 | 148 |
| ***tnf-α*** | 5’-AGCTGTAGGCCCCAGTGAGT-3’ | 5’-TGAGGAGGACGAACATCCAA-3’ | 50 | 275 |
| ***gapdh*** | 5’-TCCTCCTGTTCGACAGTCAGCCG-3’ | 5’-ACAGTTTCCCGGAGGGGCCAT-3’ | 55 | 653 |

**Table S1.** The primer sequences for reverse transcriptase-PCR.
