# Supplementary material for: Corrigendum to “Zanthoxylum ailanthoides Suppresses Oleic Acid-Induced Lipid Accumulation through an Activation of LKB1/AMPK Pathway in HepG2 Cells”
Source: Evid Based Complement Alternat Med. 2019 Jul 14;2019:3498219. doi: 10.1155/2019/3498219 (PMC6662453; doi:10.1155/2019/3498219)
Supplement: Supplementary Materials — The original Western blot bands of Figures 3 and 4. [file 3498219.f1.pptx]

## Slide 1
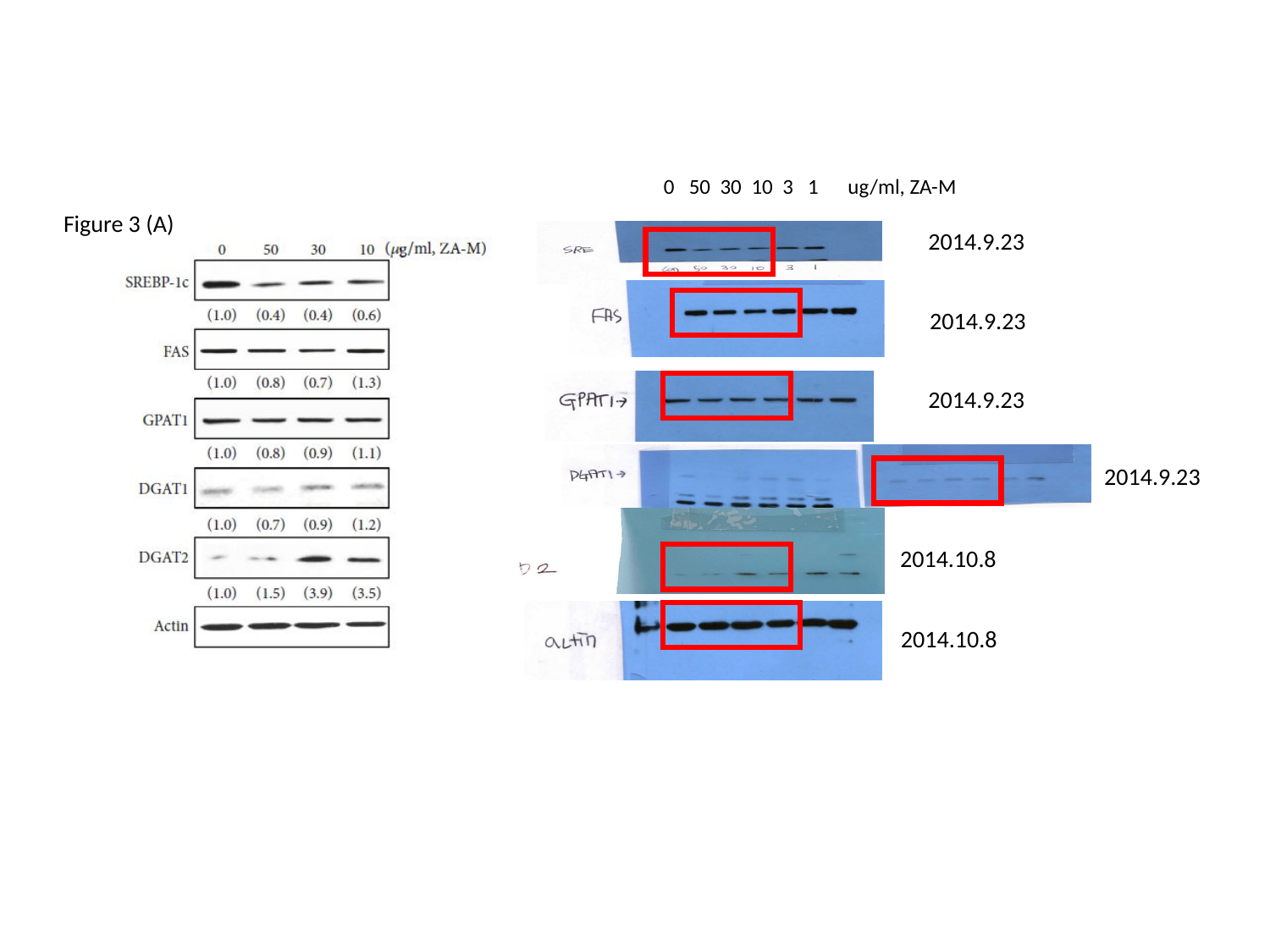

0 50 30 10 3 1 ug/ml, ZA-M
Figure 3 (A)
2014.9.23
2014.9.23
2014.9.23
2014.9.23
2014.10.8
2014.10.8

## Slide 2
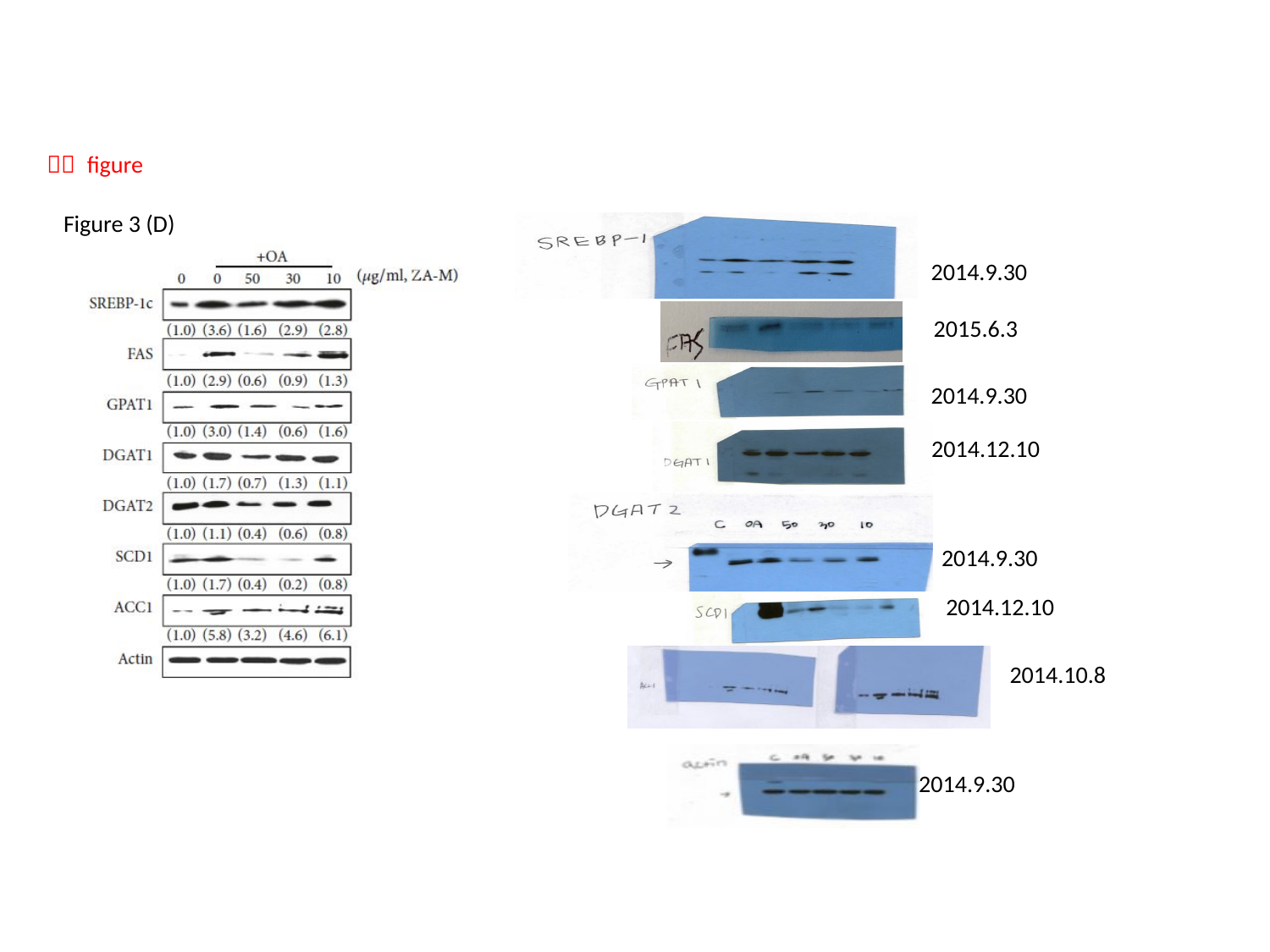

논문 figure
Figure 3 (D)
2014.9.30
2015.6.3
2014.9.30
2014.12.10
2014.9.30
2014.12.10
2014.10.8
2014.9.30

## Slide 3
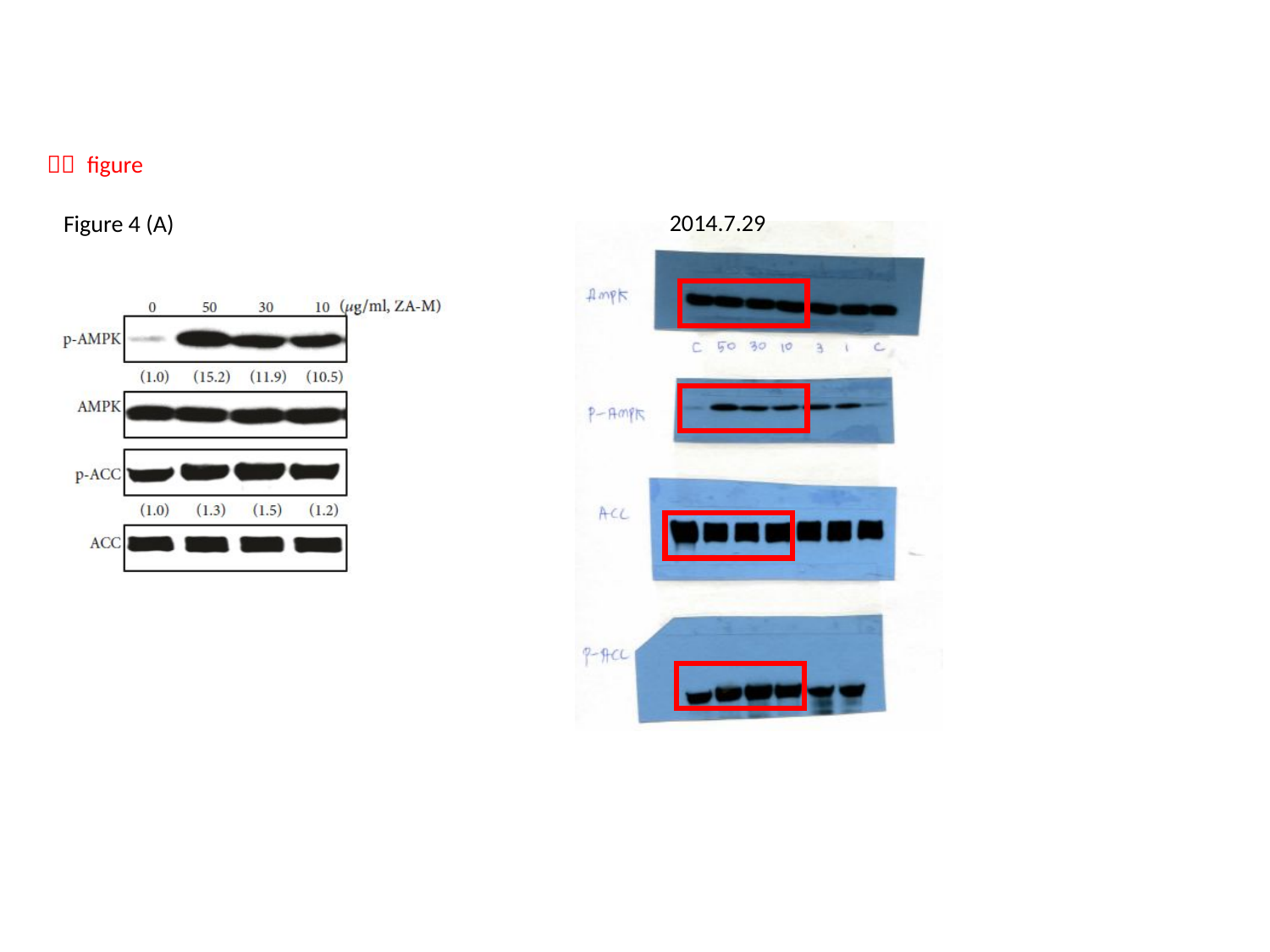

논문 figure
2014.7.29
Figure 4 (A)

## Slide 4
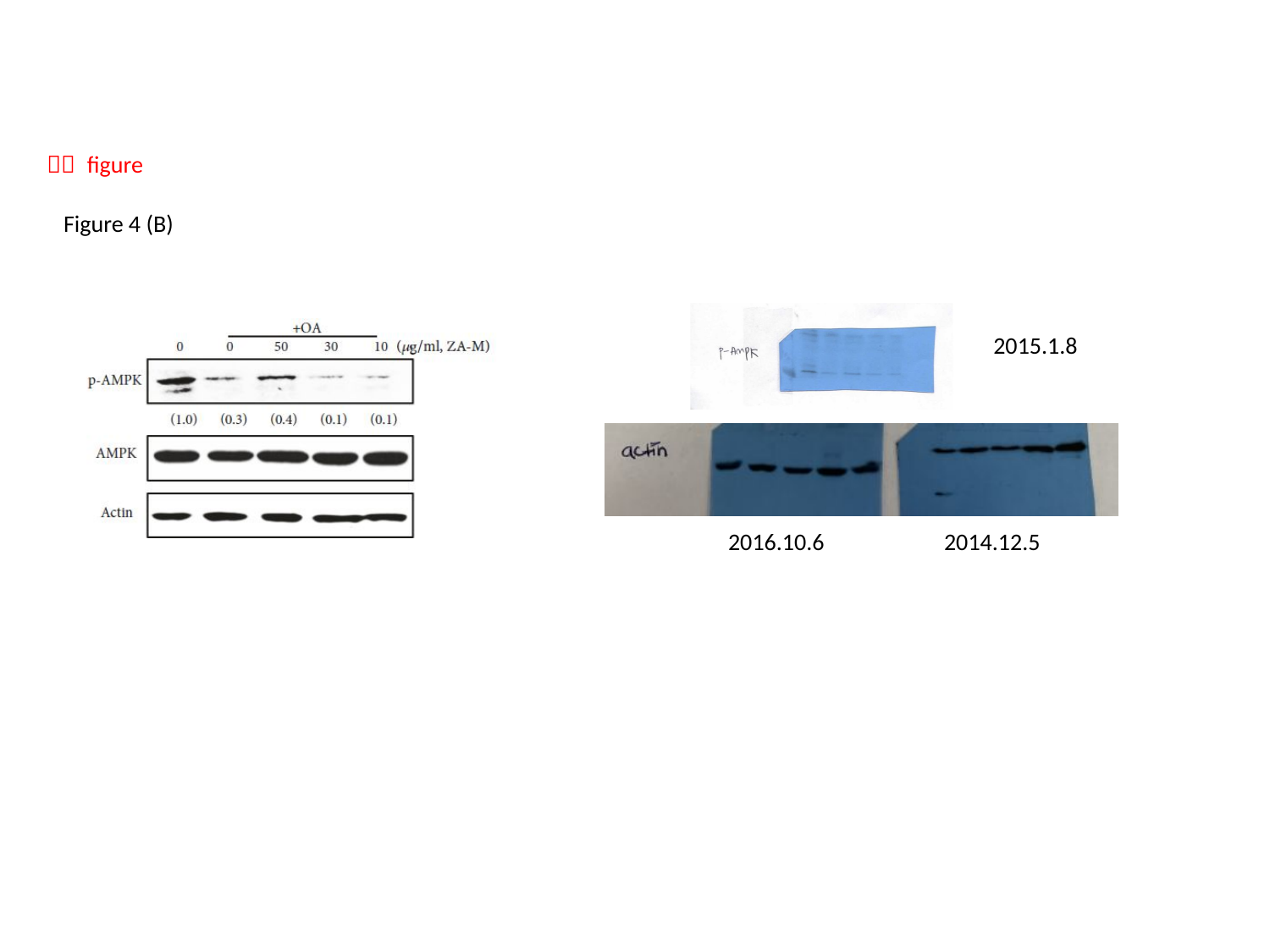

논문 figure
Figure 4 (B)
2015.1.8
2016.10.6
2014.12.5

## Slide 5
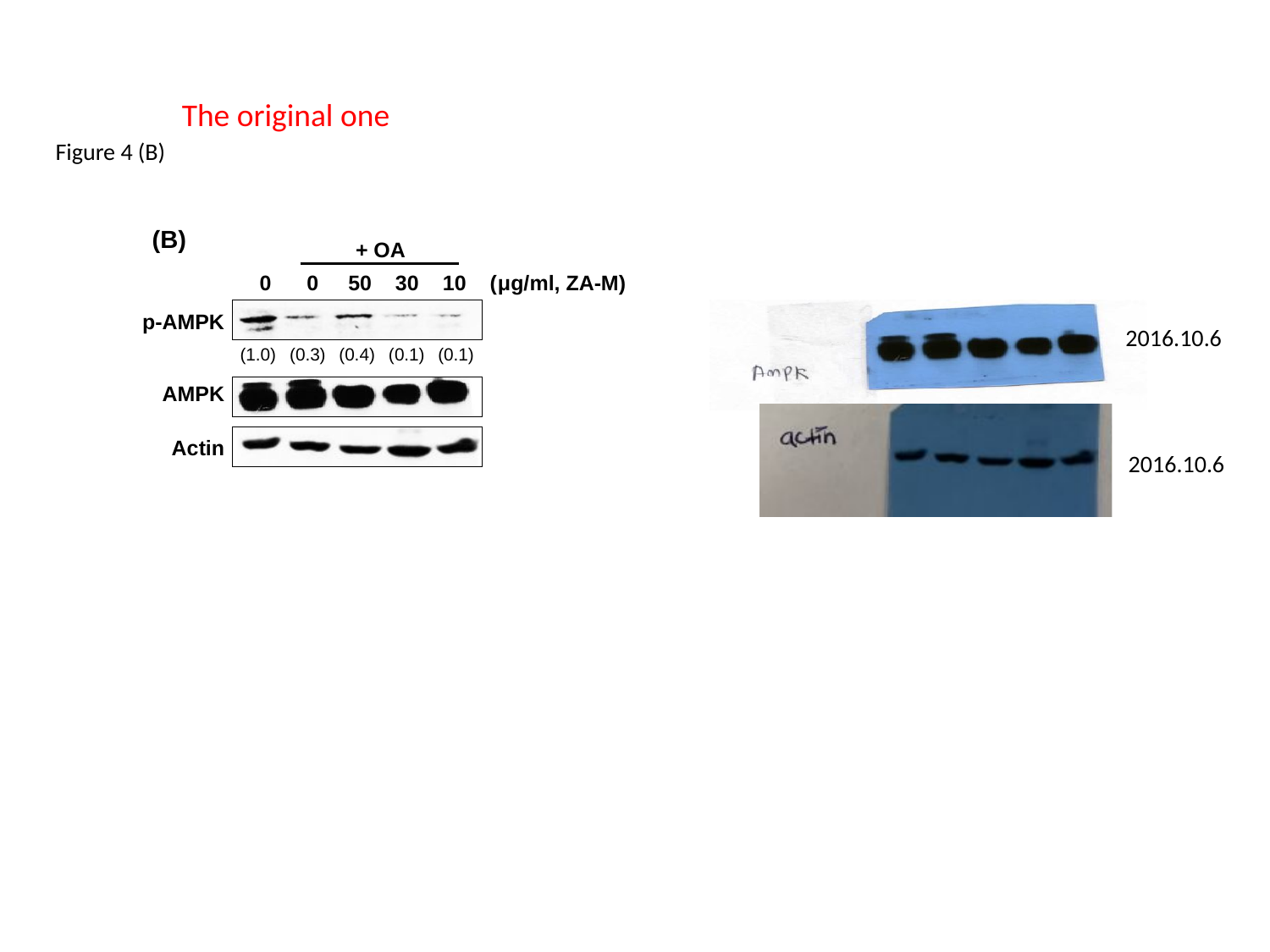

The original one
Figure 4 (B)
(B)
+ OA
0 0 50 30 10 (μg/ml, ZA-M)
p-AMPK
2016.10.6
(1.0)
(0.3)
(0.4)
(0.1)
(0.1)
AMPK
Actin
2016.10.6
